# Evaluating the Impact of Flossing Band Integration in Conventional Physiotherapy for Patellofemoral Pain Syndrome

**DOI:** 10.3390/jcm13102958

**Published:** 2024-05-17

**Authors:** Felipe León-Morillas, Manuel García-Marín, Carlota Corujo-Hernández, María Martín Alemán, Yolanda Castellote-Caballero, Lawrence P. Cahalin, Aday Infante-Guedes, David Cruz-Díaz

**Affiliations:** 1 Department of Physiotherapy, Faculty of Physiotherapy, Podiatry and Therapy Occupational, Catholic University of Murcia (UCAM), Guadalupe, 30107 Murcia, Spain; fleon@ucam.edu; 2 Department of Health Sciences, Faculty of Health Sciences, University of Jaén, 23071 Jaén, Spain; mgm00049@red.ujaen.es (M.G.-M.); dcruz@ujaen.es (D.C.-D.); 3 Grupo ICOT, 38320 La Cuesta, Spain; supervision.lacuesta@grupoicot.es; 4 Grupo ICOT, 35200 Arnao, Spain; supervision.arnao@grupoicot.es; 5 Department of Physical Therapy, University of Miami, Miller School of Medicine, Coral Gables, FL 33146, USA; l.cahalin@miami.edu; 6 Faculty of Health Sciences, University of Atlántico Medio, 35017 Las Palmas de Gran Canaria, Spain; aday.infante@pdi.atlanticomedio.es

**Keywords:** patellofemoral pain syndrome, flossing band therapy, physiotherapy, Anterior Knee Pain Syndrome, rehabilitation

## Abstract

**Background/Objectives**: Patellofemoral Pain Syndrome (PFPS) is prevalent among physically active individuals, highlighting the need for innovative treatment strategies beyond conventional physiotherapy. This study investigates the effectiveness of integrating flossing band therapy with standard physiotherapy, anticipating improved outcomes in pain reduction, functional ability, and patient satisfaction. **Methods:** A double-blinded randomized controlled trial involved 50 PFPS-diagnosed participants. They were divided into two groups: Standard Physiotherapy Group (SPG) and Flossing Band and Physiotherapy Group (FBPG), each undergoing an 8-week intervention focusing on resistance training supplemented by respective therapies. Assessment metrics included pain (VAS), strength (Dynamometry), lower limb function (LEFS), and PFPS function (AKPS) measured before and after the intervention. **Results:** Significant enhancements in all outcome measures were noted for both groups, yet the FBPG exhibited notably superior improvements in pain, knee functionality, muscle strength, and lower extremity function. The FBPG demonstrated statistically significant greater efficacy in pain alleviation and strength enhancement. **Conclusions:** The addition of flossing band therapy to conventional physiotherapy presents a more effective treatment modality for PFPS, suggesting its potential to redefine therapeutic standards. Future studies should delve into the long-term impacts and mechanistic underpinnings of floss band therapy in PFPS management.

## 1. Introduction

Patellofemoral Pain Syndrome (PFPS), often referred to as “runner’s knee,” poses a considerable clinical conundrum, attributable to its multifaceted origins and its widespread occurrence within physically active demographics. Manifesting as pain in the anterior region of the knee, particularly around the patella, PFPS is a predominant source of knee discomfort among athletes, adolescents, and young adults [1]. The intricate nature of its pathophysiology, which includes biomechanical, anatomical, and psychological components, necessitates an all-encompassing strategy for both diagnosis and treatment [2].

Patellofemoral Pain Syndrome (PFPS) is characterized by pain situated at the front of the knee, specifically near the patella or kneecap. This pain often worsens during activities that increase pressure on the knee joint, such as running, squatting, and going up or down stairs. [2]. The etiology of PFPS is recognized as multifactorial, encompassing biomechanical and anatomical factors such as aberrant patellar tracking within the femoral groove, imbalances in the musculature of the quadriceps and hip abductors, and cumulative strain injuries. Moreover, the syndrome is often accompanied by symptoms such as a sensation of grinding or clicking within the knee joint, signaling potential chondromalacia patellae, which involves the softening and degeneration of cartilage beneath the kneecap [1,2,3]. Emerging evidence also underscores the critical role of neuromuscular control and proprioception in the onset of PFPS [2].

PFPS ranks as one of the most prevalent knee disorders, with a higher incidence among females, possibly due to anatomical and hormonal variances affecting knee biomechanics. Epidemiological research has pinpointed numerous risk factors for PFPS, including functional muscular weakness, tight musculature, general ligamentous laxity, and biomechanical abnormalities such as foot overpronation and dysfunction of hip musculature [4,5,6]. Sports that impose significant knee impacts, such as running, basketball, and soccer, frequently report this condition [2]. Furthermore, the likelihood of developing PFPS is modulated by variables like training frequency, intensity, the nature of surfaces, and the type of footwear used [1]. Recent statistics reveal notable prevalence rates, with annual percentages in the general populace and among adolescents estimated at 22.7% and 28.9%, respectively, underscoring the escalating concern regarding PFPS in both athletic and non-athletic demographics [4].

Physiotherapy stands as a fundamental treatment for PFPS. The therapeutic approach typically includes exercises aimed at strengthening the quadriceps, hamstrings, and hip muscles, as well as stretching and mobility exercises to improve joint function [7,8]. Evidence from clinical studies suggests that these physiotherapeutic interventions significantly reduce pain and enhance knee function in PFPS patients [7]. The effectiveness of physiotherapy in PFPS management has been recognized as superior to other conservative treatments, such as rest, knee braces, or NSAIDs, particularly in the long-term management of the condition [7,8]. Additionally, modalities like patellar taping and foot orthoses have been shown to provide immediate pain relief and improve functional outcomes [9]. Recent advancements in physiotherapy include flossing band therapy. Flossing application is a relatively recent technique involving compression bandaging typically for limb joints and has garnered attention in the realm of knee pathology treatment. Previous research reported that floss band application can significantly improve joint range of motion and reduce pain in individuals with knee arthritis [10], knee extensor maximum voluntary contraction torque [11], and significantly reduce perceived pain among athletes [12]. However, although flossing band therapy shows promise for knee pathology, evidence suggests that its long-term effectiveness remains to be conclusively determined [13,14].

Our study hypothesizes that incorporating flossing band therapy into conventional physiotherapy for patients with Patellofemoral Pain Syndrome (PFPS) will result in more significant improvements in pain reduction, functional ability, and overall patient satisfaction compared to conventional physiotherapy alone. This hypothesis is predicated on the potential of flossing bands to enhance blood flow, reduce muscular tightness, and improve joint mobility, thereby augmenting the efficacy of standard physiotherapeutic techniques [11,12,14]. 

This research aims to bridge a critical gap in the current PFPS treatment paradigm. By methodically evaluating the efficacy of adding flossing band therapy to conventional physiotherapy, the study endeavors to provide empirical evidence for a potentially more effective treatment approach. The findings are expected to inform physiotherapy practices, offering clinicians a novel, evidence-based method to enhance PFPS management, ultimately improving patient outcomes in both the short and long term. 

## 2. Materials and Methods

### 2.1. Study Design

A double-blinded, randomized controlled trial was conducted to evaluate the influence of adding flossing band application to conventional physiotherapy based on 8 weeks of resistance training in patients with PFPS. Dependent variables were Pain (VAS), Strength (Dynamometry), Lower Limb Function (LEFS), and PFPS Function (AKPS), and independent variables were time (pre and post-intervention) and group (conventional physiotherapy vs. flossing band application). This research project was approved by the research ethics committee of the University of Jaén and was conducted in accordance with the Code of Ethics of the World Medical Association for Human Studies (Declaration of Helsinki). This work was registered in the online database: Clinicatrail.gov, managed by the National Library of Medicine of the United States, under registration number: NCT06271811.

### 2.2. Participants

**Figure 1:**
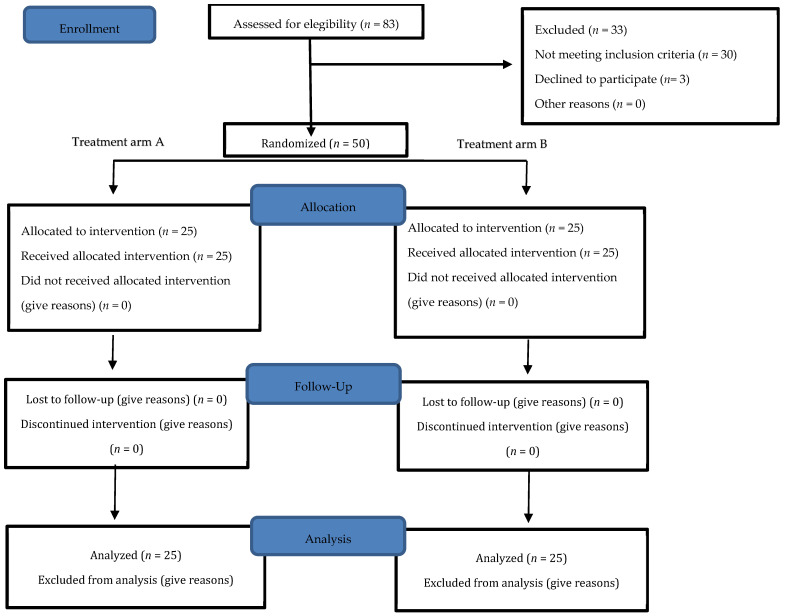
Flow chart of the study design and participants follow up through the trial.

A sample of 50 participants diagnosed with Patellofemoral Pain Syndrome (PFPS) were randomly allocated into two distinct groups to assess the effectiveness of different therapeutic approaches. The control group, termed the Standard Physiotherapy Group (SPG, *n* = 25), adhered to a rehabilitation program in line with current PFPS treatment guidelines, primarily focusing on neuromuscular intervention. In contrast, the intervention group, named the Flossing Band and Physiotherapy Group (FBPG), underwent a combined treatment regimen of conventional physiotherapy, as practiced in the SPG, along with additional flossing band therapy targeting the knee region to augment the therapeutic effects through compression and mobilization techniques. (Figure 1).

To be eligible for inclusion in this study, subjects must meet the following criteria based on previous research [15]:

### 2.3. Inclusion Criteria

- An age range of 18 to 50 years, to include a broad spectrum of the population affected by PFPS.
- Experience retro patellar pain for at least three months, including pain at rest or during activities such as ascending or descending stairs, jumping, running, squatting, kneeling, or prolonged sitting.
- Pain or apprehension upon mobilization of the patella and/or crepitus accompanied by pain during activities like squats.
- No history of medical treatment, physiotherapy specifically targeting PFPS, or lower extremity surgery within the last six months.
- Exclusion Criteria:
- A history of significant lower extremity, pelvic, or spinal surgery/fracture, or traumatic lesions of ligaments or meniscus within the past six months.
- Presence of other orthopedic conditions (e.g., ACL rupture, meniscal tears), neurological disorders (e.g., multiple sclerosis, paralysis), rheumatological diseases (e.g., rheumatoid arthritis, ankylosing spondylitis), or congenital conditions leading to osteoarthrosis.
- Current pregnancy.
- A history of connective tissue disease, patellofemoral dislocation or subluxation, or osteoarthrosis of the knees.
- Use of sedatives or muscle relaxants that may alter muscle tone.
- Other forms of anterior knee pain (e.g., Osgood-Schlatter disease, tendon pain, bursitis).
- A history of referred pain from the lumbar spine.

Participants, encompassing a diverse gender and age spectrum, were assessed at baseline and after 8 weeks of undergoing their respective rehabilitation protocols. Standardized measures of pain intensity, knee functionality, and overall mobility were employed for these assessments.

To ensure methodological rigor, an independent researcher, not involved in participant recruitment, evaluations, or the rehabilitation process, was responsible for the random allocation of participants to either group, maintaining a 1:1 ratio. Furthermore, the evaluators responsible for assessing the treatment outcomes were blinded to the group assignments of participants. This blinding protocol was also applied to the physical therapists administering the rehabilitation programs, who remained unaware of the specific group evaluations and outcomes.

The sample size was determined through power analysis using the GPower 3.0.10 software. based on the 13-point difference in the Anterior Knee Pain Score (AKPS), with a standard deviation of 12.8 and 0.80 power with a level of significance a = 0.05 with two-tailed *t*-test, and an estimated drop out of 15% [16]. A minimum sample size of 23 subjects per group was indicated.

### 2.4. Outcome Measures

*Disability-related to patellofemoral pain.* The Kujala Anterior Knee Pain Scale (AKPS), developed by Kujala et al. [17], This self-administered questionnaire, a recognized instrument for assessing symptoms and functional impairments in individuals with Patellofemoral Pain Syndrome (PFPS), consists of 13 questions. Responses are scored to give a total that can range from 0 to 100, with higher values indicating superior knee function and fewer symptoms. Although there are no universally agreed-upon thresholds, scores approaching 100 typically suggest minimal issues related to patellofemoral pain. The questionnaire is acclaimed for its dependability and accuracy, as evidenced by numerous studies confirming its consistent internal structure and excellent test-retest reliability. This makes it an effective tool for both initial evaluations and ongoing tracking of patient progress. [18]. Its validity is further supported by its sensitivity to treatment interventions, rendering it a valuable instrument in clinical and research settings for evaluating the impact of PFPS [19].

*Knee extensor muscle strength.* Muscle strength of the knee extensor muscles in patients diagnosed with Patellofemoral Pain Syndrome (PFPS) was assessed using a handheld dynamometer in a 90° fixed position. The measurement that demonstrated the greatest force output was selected for subsequent analysis. Handheld dynamometry exhibits a moderate to strong correlation with isokinetic dynamometry, along with satisfactory reliability between different testers and excellent reliability in repeated tests for measuring maximal voluntary contraction (MVC) of the quadriceps and hamstrings [20,21]. To allow the patient to monitor their progress and incorporate the test results into a strength protocol based on 1 RM% calculation, values were recorded in kilograms instead of newtons. 

*Pain report.* Pain levels were assessed using a visual analog scale (VAS). This scale features a 10-cm line where the left end signifies no pain and the right end represents the most severe pain imaginable. Participants marked their present pain intensity on this line, with higher markings corresponding to greater pain. The VAS is considered a dependable method for monitoring pain in patients suffering from Patellofemoral Pain Syndrome (PFPS). [22].

*Self-reported function.* The functional status of the lower limbs was evaluated using the Lower Extremity Functional Scale (LEFS). This instrument, noted for its validity and reliability, is used to assess musculoskeletal dysfunction in the lower extremities, whether acute or chronic [23]. Comprising 20 items, each item on the LEFS is rated on a scale from zero to four, reflecting the difficulty in performing specific activities due to physical impairment. The overall scores are summed to a total between 0 and 80, where lower scores represent decreased functionality and higher scores indicate greater functional capacity. The LEFS has been validated as a reliable self-reported measurement tool for assessing lower limb function. [23,24].

### 2.5. Intervention

#### 2.5.1. Conventional Physiotherapy

The treatment protocol focuses on neuromuscular training, primarily aimed at fortifying essential muscle groups such as the quadriceps, hamstrings, and hip abductors, which are crucial for stabilizing the knee. This strategy is underpinned by previous studies [25] that highlight the significance of these muscles in maintaining knee joint health. Additionally, the regimen includes proprioceptive exercises like single-leg stands and controlled knee flexions to improve the sense of joint positioning. These exercises are in accordance with guidelines proposed by Powers et al., 2010 [26]. The intensity of these exercises is progressively increased, tailored to each patient’s tolerance and improvement. The protocol stipulates two sessions per week over eight weeks.

#### 2.5.2. Flossing Band Group Protocol

**Figure 2:**
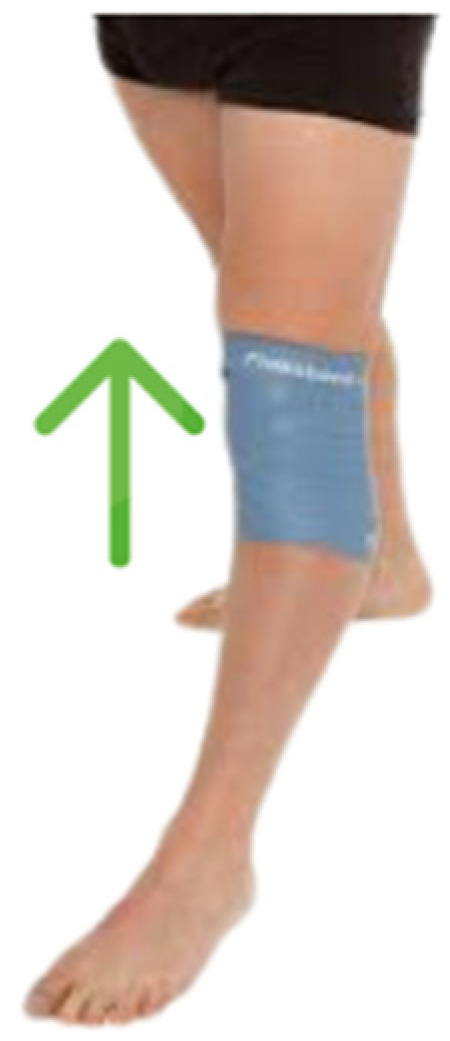
Flossing band application.

In addition to the aforementioned protocol, participants in the flossing band group receive a treatment that includes the application of a flossing band. The flossing band, made from a thick elastic latex, is 1.3 mm thick, 50 mm wide, and 2 m long, with a strength level of 3 (COMPRE Floss, Sanctband, Hamburg, Germany). Before each physiotherapy session, the flossing band is applied around the knee joint to provide moderate compression. The application of flossing to the knee commences with a preliminary evaluation of the joint range of motion and pain perception. The bandaging technique was meticulously applied by the principal investigator, who was also responsible for its removal. The procedure was initiated approximately 2–3 cm below the inferior pole of the patella, with the band being placed in an upward direction around the thigh. Each subsequent layer of the band overlapped the previous by about 25 mm. During this process, the patient was positioned in an upright stance without fully locking the knee. The final portion of the band was securely fastened below the preceding wrap. The band is wrapped around the knee, starting with an approximate tension of 50% for the first wrap and escalating to a tension of 60–80% in subsequent wraps. It is essential to overlap the band with each wrap, moving from a distal to the proximal direction (bottom to top), which aids in enhancing drainage. (Figure 2). Upon application of the bandage, the participants executed the exercises as specified in the preceding protocol. 

Continuous monitoring of the patient is critical to ensure that excessive pressure is not being applied. This can be achieved by palpating the pulse on the dorsum of the foot or inner ankle and observing if the skin regains its normal color after pressing the area with fingers. Should the patient experience strong tingling or pain, the bandage must be immediately removed. The duration of the band’s application varies between 2 to 5 min, depending on patient tolerance, a methodology informed by existing research [27]. Participants in both groups were advised not to alter their usual activity nor to participate in additional training programs beyond those of the intervention.

After the initial application of the flossing band during joint mobilizations, it is removed, allowing patients to engage in active neuromuscular training exercises. The session concludes with a re-application of the flossing band for an additional 2–5 min. This final application is aimed at supporting recovery and minimizing any post-exercise discomfort.

### 2.6. Statistical Analysis

To compare the variables between groups, the Student’s t-test or non-parametric equivalent, the Mann–Whitney U test, was used. To assess differences in strength, VAS, LEFS, and AKPS (dependent variables) between evaluation times within groups, 2 groups by 2 times ANOVA with repeated measures or the non-parametric alternative, the Friedman test, were used. Chi-square was used to compare the descriptive data of the participants. Furthermore, to carry out the study within-group change scores, confidence intervals have been calculated for the difference in means for paired or dependent samples, hypothesis tests for two dependent or paired samples, and Cohen’s D. Statistical analysis was performed using the SPSS software version 28.0 (SPSS Inc., Chicago, IL, USA). The α level was set at *p* < 0.05 for all tests.

## 3. Results

**Figure 3:**
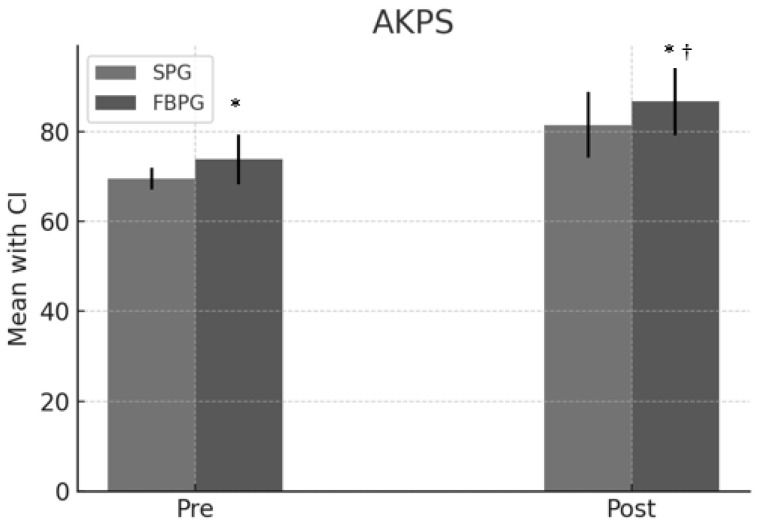
Kujala Anterior Knee Pain Scale (AKPS). * Statistical difference pre-post intervention. † Statistical difference between groups.

Out of the initial 83 patients who participated in the recruitment process, 50 individuals met the eligibility criteria, completed the study, and were included in the analysis. The sociodemographic characteristics of the sample were collected at baseline, and no significant differences were observed between groups. (Table 1). Statistical analysis revealed significant differences between intervention groups across several variables (Table 2). In the analysis of the Kujala Anterior Knee Pain Scale (AKPS), assessing knee pain and functionality, both groups showed improvements, with the SPG increasing from 69.5 [CI: 67.1, 71.9] to 81.5 [CI: 77.1, 91.9] and the FBPG from 73.8 [CI: 67.2, 78.4] to 86.8 [CI: 75.2, 90.4]. The mean difference between the groups was 5.3 [CI: 4.42, 6.18], with a *p*-value of 0.001 and an effect size of 2.08, favoring the FBPG. (Figure 3).

**Figure 4:**
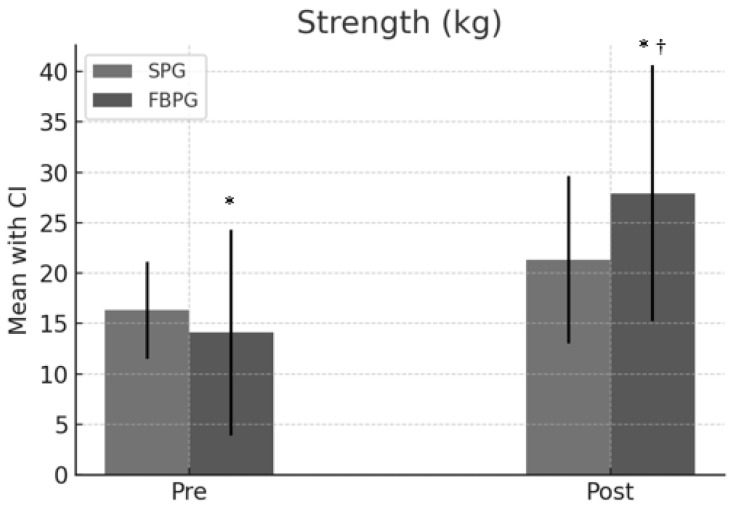
Knee extensor Strength pre and post-intervention. Pre and post-intervention. * Statistical difference pre-post intervention. † Statistical difference between groups.

Regarding strength, measured in kilograms, significant improvements were observed in both groups. The SPG improved from an average of 16.3 [CI: 15.5, 25.1] to 21.3 [CI: 18.5, 35.1], and the FBPG from 14.1 [CI: 12.2, 32.6] to 27.9 [CI: 21.2, 46.6]. The mean difference between the groups was 6.6 [CI: 5.72, 7.48] in favor of the FBPG, with a *p*-value of 0.001 and an effect size of 3.22, suggesting significant superiority of FBPG in strength improvement. (Figure 4).

In the analysis of the Visual Analogue Scale (VAS) for pain, both groups demonstrated a significant reduction in post-intervention scores compared to pre-intervention. The SPG decreased from an average of 5.8 [CI: 4.9, 6.7] to 2.8 [CI: 1.4, 5.1], while the FBPG decreased from 5.2 [CI: 3.7, 5.9] to 1.8 [CI: 1.1, 3.9]. The mean difference between groups post-intervention was −1.0 [CI: −1.88, −0.12], with a *p*-value of 0.001 and a Cohen’s effect size of 0.64, indicating greater efficacy of FBPG in reducing pain. (Table 2).

Finally, for the Lower Extremity Functional Scale (LEFS), both groups experienced improvements post-intervention. The SPG increased from 37.7 [CI: 28.2, 45.2] to 62.7 [CI: 60.2, 65.2], and the FBPG from 39.1 [CI: 27.6, 45.8] to 69.2 [CI: 67.6, 70.8]. The mean difference was 6.5 [CI: 5.62, 7.38], with a *p*-value < 0.001 and an effect size of 2.13, indicating greater improvement in the FBPG. (Table 2).

In conclusion, the results suggest that although both treatments were effective, the Flossband and Physiotherapy Group (FBPG) demonstrated greater efficacy in reducing pain and improving strength, lower extremity function, and knee functionality compared to the Standard Physiotherapy Group (SPG). These findings are supported by significant *p*-values and substantial effect sizes.

## 4. Discussion

The results of our study contribute to the evolving understanding of Flossing as a therapeutic modality for patellofemoral pain syndrome (PFPS). Our findings indicate that a physiotherapy approach based on strength training, both with and without the addition of Flossing, significantly reduces pain and improves functionality in individuals with PFPS. However, the group that received Flossing in conjunction with physiotherapy intervention exhibited superior outcomes, suggesting a synergistic effect that merits further exploration.

Flossing, also known as compression band therapy, is grounded in the principle of applying pressure to musculoskeletal areas to elicit physiological changes that can facilitate recovery and improved function. The physiological mechanisms through which Flossing exerts its effects are multifaceted. The application of the Flossing band has been deemed to induce changes in fascial elasticity, which is essential for maintaining the sliding and gliding of tissues necessary for joint movement [28]. This compression may temporarily restrict blood flow to the area, which, upon release, encourages a surge of fresh blood that can help in the removal of waste products and the delivery of nutrients to the tissues [29].

Furthermore, regarding strength improvement, our analysis suggests that the application of Flossing has a positive effect on enhancing muscle strength in this population when compared to the conventional physiotherapy group. Our findings agree with those reported by Kaneda et al. [30], who observed improvements in hamstring muscle function following Flossing. This suggests that Flossing may facilitate gains in muscle strength, which is crucial for the rehabilitation of Patellofemoral Pain Syndrome (PFPS). Chang et al. [31] further support the potential of Flossing to enhance muscle force production and flexibility, both essential components of functional recovery in PFPS patients.

The influence of flossing on pain management appears to yield promising results, as evidenced by the findings obtained in our study. The addition of Flossing to strength training was associated with improved outcomes in pain reduction and limb functionality. This is in line with the findings of Driller et al. [32], who reported enhanced ankle dorsiflexion following Flossing. Similarly, García-Luna et al. [33] found that Flossing led to improved vertical jump performance and a significant reduction in perceived knee pain, which supports the notion that Flossing can positively affect both performance and pain perception. Our results may be explained by considering that myofascial compression provided by Flossing is thought to facilitate joint distraction, allowing for mobilization techniques with reduced tension and discomfort. This can enhance the sliding of fascial layers over one another, potentially improving joint range of motion and reducing pain [34]. Moreover, the pressure exerted by the band can stimulate mechanoreceptors and free nerve endings, which may modulate pain and enhance proprioceptive feedback [35].

In the context of PFPS, our study suggests that Flossing may offer additional benefits over strength training alone. The physiological effects of Flossing, such as improved blood flow, fascial mobilization, and enhanced proprioception, may contribute to these outcomes [28,31]. The superior improvements observed in the Flossing group underscore the potential of this modality as a valuable adjunct to conventional strength training in the management of PFPS. However, it is important to note that the literature presents mixed findings regarding the efficacy of Flossing. Hadamus et al. [36] reported no significant changes in muscle strength parameters with Flossing, indicating that the effects may vary based on individual factors or the specific protocols employed. This highlights the need for personalized treatment plans and further research to delineate the optimal use of Flossing in different populations and conditions.

Physical therapy has demonstrated its effectiveness in enhancing functional outcomes and alleviating pain in individuals with patellofemoral syndrome [37]. Notably, strength training, a fundamental component of physiotherapy, is recognized as a cornerstone in the comprehensive management of this condition [38]. In our study, the superior outcomes associated with the application of Flossing, as compared to a strength-focused approach, can be attributed to its potential advantages, which encompass enhanced circulation, inflammation reduction, and alleviation of tissue congestion [28,31]. Additionally, the influence of flossing on kinesiophobia, a common psychological aspect accompanying PFPS, may play an important role. Gradually reintroducing pain-free movement through a combination of strength training and flossing may help reduce fear of movement, ultimately leading to enhanced overall functionality and symptom relief [39]. This dual-pronged approach, encompassing both physiological and psychological dimensions, offers a comprehensive solution for individuals managing the patellofemoral syndrome.

Although the results from the application of the flossing band seem promising, and its use is straightforward and apparently safe, it is essential to consider several factors. These include the potential influence of the material type, the resistance of the patient to the applied pressure during bandaging, and the potential degradation of the material’s properties. Importantly, the application of pressure, though not deemed critical, depends on the subjective sensation experienced by both the patient and the therapist. Furthermore, the study did not include an evaluation of the patient’s range of motion before and after the intervention, both in their physiological range and during functional tasks, which represents a significant limitation. It is recommended that future studies take into account these considerations in order to determine the most effective method of applying this novel treatment.

## 5. Conclusions

The findings of this study substantiate the integration of flossing band therapy as a complementary treatment within the realm of conventional physiotherapy for managing Patellofemoral Pain Syndrome (PFPS). It is noteworthy that flossing band therapy has demonstrated a commendable safety profile, with no adverse effects reported, thereby affirming its suitability for clinical application. The investigation reveals that flossing band therapy appears to potentiate the therapeutic effects of standard physiotherapy interventions. This enhancement in treatment efficacy suggests that flossing band therapy could substantially improve patient outcomes in pain alleviation and functional recovery.

Furthermore, the application of the flossing band is characterized by simplicity and cost-effectiveness. This treatment does not necessitate elaborate resources nor significant financial outlays, facilitating its adoption across a broader spectrum of clinical settings. It is also proposed that with appropriate instruction, patients could be empowered to apply flossing bands independently. Such a practice would enable patients to continue and complement their treatment autonomously, potentially improving long-term outcomes and patient satisfaction. The rapid application of the flossing band and its effortless integration into established physiotherapeutic protocols underscore its viability and efficacy as a treatment option within clinical settings.

## Figures and Tables

**Table 1:** Demografic charasteristics at baseline.

		**SPG (*n* = 25)**	**FBPG (*n* = 25)**	***p* Value**
Age		48.63 ± 10.27	46.58 ± 11.89	0.44
Gender %				0.87
	Male	8 (32%)	6 (24%)	
	Female	17 (68%)	19 (76%)	
Height (cm)		169 ± 9.7	167 ± 7.2	0.62
Weight (Kg)		64.73 ± 9.12	66.02 ± 8.63	0.32
Education				0.63
	Primary	3 (12%)	1 (4%)	
	Secondary	10 (40%)	12 (48%)	
	University	12 (48%)	12 (48%%)	
Marital Status				0.61
	Single	3 (12%)	6 (24%)	
	Married	16 (64%)	16 (64%)	
	Divorced	6 (24%)	3 (12%)	
Occupational status				0.28
	Full-time Worker	19 (76%)	17 (68%)	
	Part-time worker	6 (24%)	5 (20%)	
	Unemployed		3 (12%)	

aOne-way analysis of variance for continuous variables and χ^2^ test for categorical variables. Standard Physiotherapy Group (SPG); Flossing Band and Physiotherapy Group (FBPG).

**Table 2:** Between and within group comparison pre and post-treatment.

		SPG (n = 25)	FBPG (n = 25)			Effect Size (Cohen’s D)
Variable		Mean (95% IC)	Mean (95% IC)	Mean Differences and (95% CI) between Groups Post-Intervention	*p*-Value	SPG vs. FBPG
VAS	Pre	5.8 [4.9, 6.7]	5.2 [3.7, 5.9]		0.163	
	8 weeks	2.8 [1.4, 5.1]	1.8 [1.1, 3.9]	−1.0 [−1.88, −0.12]	0.001	0.64
Strength (kg)	Pre	16.3 [15.5, 25.1]	14.1 [12.2, 32.6]		0.125	
	8 weeks	21.3 [18.5, 35.1]	27.9 [21.2, 46.6]	6.6 [5.72, 7.48]	0.001	3.22
LEFS	pre	37.7 [28.2, 45.2]	39.1 [27.6, 45.8]		0.17	
	8 weeks	62.7 [60.2, 65.2]	69.2 [67.6, 70.8]	6.5 [5.62, 7.38]	<0.001	2.13
AKPS	pre	69.5 [67.1, 71.9]	73.8 [67.2, 78.4]		0.09	
	8 weeks	81.5 [77.1, 91.9]	86.8 [75.2, 90.4]	5.3 [4.42, 6.18]	0.001	2.08

Standard Physiotherapy Group (SPG); Flossing Band and Physiotherapy Group (FBPG); VAS (visual analog Scale); Strenght kilograms (Kg); Lower Functional Scale (LEFS); Kujala Anterior Knee Scale (AKPS).

## Data Availability

The data presented in this study are available at the request of the corresponding author due to data protection policies.
